# Estimation of 3D Body Center of Mass Acceleration and Instantaneous Velocity from a Wearable Inertial Sensor Network in Transfemoral Amputee Gait: A Case Study

**DOI:** 10.3390/s21093129

**Published:** 2021-04-30

**Authors:** Emeline Simonetti, Elena Bergamini, Giuseppe Vannozzi, Joseph Bascou, Hélène Pillet

**Affiliations:** 1 INI/CERAH, 47 Rue de l’Echat, 94000 Créteil, France; joseph.bascou@invalides.fr; 2 Institut de Biomécanique Humaine Georges Charpak, Arts et Métiers, 151 Boulevard de l’Hôpital, 75013 Paris, France; helene.pillet@ensam.eu; 3 Department of Movement, Human and Health Sciences, Interuniversity Centre of Bioengineering of the Human Neuromusculoskeletal System, University of Rome “Foro Italico”, Piazza Lauro de Bosis 15, 00135 Roma, Italy; elena.bergamini@uniroma4.it (E.B.); giuseppe.vannozzi@uniroma4.it (G.V.)

**Keywords:** sensor network, wearable sensors, gait analysis, lower-limb amputation, CoM, prosthesis, locomotion, MIMU, kinematics

## Abstract

The analysis of the body center of mass (BCoM) 3D kinematics provides insights on crucial aspects of locomotion, especially in populations with gait impairment such as people with amputation. In this paper, a wearable framework based on the use of different magneto-inertial measurement unit (MIMU) networks is proposed to obtain both BCoM acceleration and velocity. The proposed framework was validated as a proof of concept in one transfemoral amputee against data from force plates (acceleration) and an optoelectronic system (acceleration and velocity). The impact in terms of estimation accuracy when using a sensor network rather than a single MIMU at trunk level was also investigated. The estimated velocity and acceleration reached a strong agreement (ρ > 0.89) and good accuracy compared to reference data (normalized root mean square error (NRMSE) < 13.7%) in the anteroposterior and vertical directions when using three MIMUs on the trunk and both shanks and in all three directions when adding MIMUs on both thighs (ρ > 0.89, NRMSE ≤ 14.0% in the mediolateral direction). Conversely, only the vertical component of the BCoM kinematics was accurately captured when considering a single MIMU. These results suggest that inertial sensor networks may represent a valid alternative to laboratory-based instruments for 3D BCoM kinematics quantification in lower-limb amputees.

## 1. Introduction

During the rehabilitation of people with lower-limb amputation, monitoring the kinematics of the body center of mass (BCoM) or the 3D ground reaction forces may reveal crucial information related to gait impairment [1,2,3]. Indeed, 3D BCoM motion has been shown to provide insight on dynamical stability [4,5,6], gait energetics [7,8,9] and gait asymmetries [1,10], in particular in this population. The gold standard methods to derive 3D BCoM motion rely on force plates and/or optical motion capture systems (OMCSs). Force plates allow the direct retrieval of BCoM acceleration through the measurement of the external forces applied on the body and the application of Newton’s second law [3]. While this method does not rely on any assumption regarding the body or its inertial properties, integration constants must be determined to obtain the BCoM velocity or displacement, which clearly impacts the accuracy of the results [2]. On the other hand, OMCSs allow tracking the positions of body-worn markers. Therefore, the application of the segmental analysis method, which consists in modeling the body as a chain of rigid segments with known inertial properties, allows obtaining first the trajectory of the segments’ centers of mass and, following a weighted average, that of the BCoM [11]. This method has been widely used in the literature for the estimation of BCoM displacement, velocity and acceleration, with some authors proposing optimal marker sets to facilitate the implementation of the method in the clinical field [12,13,14,15]. When this methodology is used, it is important to ensure that the selected inertial model is adapted to the population studied, as it has a significant impact on the estimated BCoM motion [2,16]. Both these methods constrain the acquisition to occur in a dedicated laboratory, which may not be adapted to clinical routine due to high system cost and complexity [17], and may result in the acquisition of a few steps only, especially when the force-plate-based method is adopted [2].

Therefore, in recent years, the use of wearable sensors has been advocated as an alternative to laboratory-based instruments for the computation of BCoM motion and ground reaction force [17,18]. In particular, magneto-inertial measurement units (MIMUs) are lightweight and low-cost sensors, embedded with tri-axial accelerometers, gyroscopes and magnetometers. These sensors measure the linear acceleration, the angular velocity and the local magnetic field along/about the axes of an inertial frame defined by the MIMU case (“MIMU local frame”). These signals can then be fused to provide an estimate of the orientation of the MIMU local frame relative to a global (Earth-fixed) reference frame [19,20]. Therefore, provided a MIMU is rigidly attached to a body segment, it might provide an estimation of its motion and thus be used for segmental analysis [21,22,23].

However, the implementation of segmental analysis with MIMUs is not as straightforward as one may think. 

First, for each MIMU-bearing segment, the acceleration is measured at the origin of the MIMU local frame and must be transferred to the segment center of mass (SCoM), which is not immediate since MIMUs do not provide an estimation of their absolute position. To overcome this limitation, some authors have proposed positioning MIMUs or accelerometers close to the underlying SCoM [22,24], which may compromise the accuracy of the retrieved accelerations. Other authors have coupled a full-body inertial model to a full-body kinematic chain [21,23]. This configuration is facilitated by commercial solutions, such as the xSens MVN suit [25] or the myoMotion software and hardware systems [26]. However, these solutions are often expensive and can be cumbersome as they require performing a rigorous sensor-to-segment calibration and impose the use of sensors on each segment pertaining to the kinematic chain. Other authors have suggested using OMCSs [27] or photographs [28] to initialize the absolute position of MIMUs with respect to the relevant SCoMs.

Second, the obtained SCoM accelerations are expressed in the MIMU local frames and must therefore be fused in a consistent global reference frame before computing the BCoM acceleration. However, the global frames sensed by several MIMUs may not be consistent across MIMUs [29,30,31], which might lead to errors when fusing data from multiple sensors. To correct for this global frame inconsistency, several authors have suggested using OMCSs [27,31,32] or photographs [30] in order to compute, for each MIMU, the orientation of its self-sensed global frame in a consistently defined global reference frame. Alternatively, a recent study has proposed using hypotheses on the orientation of segments during a static posture and sensor-to-segment calibration procedures to correct the global frames sensed by each sensor [33].

In order to facilitate MIMU-based segmental analysis in the clinical field, it is essential to keep the number of required sensors as low as possible while achieving sufficient accuracy [14,17]. Therefore, similarly to what is done with OMCSs, the sacral method paradigm, consisting of using a single sensor positioned on the lower back, has been widely investigated [34,35,36,37,38,39]. While this approach is quick and easy to implement, it was shown to lack accuracy when dynamical motion of the upper body was involved [2,37,39] or in case of asymmetrical gait pattern [15,35], such as for people with lower-limb amputation [40,41]. Some authors have therefore proposed optimal sensor networks in order to limit the number of segments instrumented with MIMUs for the computation of BCoM-derived parameters [39,42,43]. To identify the optimal location and number of sensors, several approaches were used: Zijlstra and coworkers instrumented the trunk and pelvis segments based on their higher mass compared to the other body segments [43], Najafi and coworkers instrumented the shank, thigh and trunk based on the observation that the motion studied (golf swing) involved mainly rotations around the ankle and hip joint [39] and, lastly, Shahabpoor and coworkers have proposed identifying the optimal location of sensors by analyzing the contributions of the individual accelerations of each SCoM in the total BCoM acceleration [24]. This last methodology is of particular interest since it is the only one applied for gait and since the MIMU-bearing segments are chosen both based on their mass and their motion. Consequently, it has been recently applied to the gait of ten people with transfemoral amputation and has shown that instrumenting the trunk, thighs and shanks or feet allows an accurate estimation of BCoM acceleration [44]. However, in this study, MIMU data were simulated using an OMCS in order to overcome the two above-mentioned issues related to reference frame consistency and absolute position. The validation of the obtained optimal sensor networks in the same population, i.e., people with transfemoral amputation, using data from actual MIMUs remains to be performed.

Another challenge when using MIMUs rather than OMCSs is the inherent noise in the sensor signals, which may lead to drift when integrating the acceleration to compute the instantaneous velocity—or displacement—of the BCoM. A solution to mitigate the drift is to express the instantaneous velocity over a gait cycle as the sum of a cyclical term and an average velocity of progression, computed from the stride length divided by the stride duration [38,45].

In light of all these considerations, this work aimed at proposing a wearable framework allowing the estimation of both the BCoM acceleration and instantaneous velocity from an optimal network of MIMUs. Several networks of MIMUs were investigated based on the results obtained in ten people with transfemoral amputation using an OMCS in a previous work [44]. As a proof-of-concept, the framework implemented in one person with transfemoral amputation is presented. Its accuracy was validated against force platforms (BCoM acceleration) and optical motion capture data (BCoM acceleration and velocity). The following criteria were taken into account in the development and validation of the proposed wearable-based framework: (1) setup and acquisition durations should be as short as possible, with a minimum number of sensors; (2) calibration procedures and processing complexity should be kept at a minimum. Compliance with these criteria is assumed to facilitate the future transfer of the proposed framework to the clinical field.

## 2. Materials and Methods

### 2.1. Implementation of a Wearable Framework

In order to estimate BCoM acceleration and velocity using an optimal sensor network of segment-mounted MIMUs, a wearable framework was implemented, consisting of the three following steps:

- Computation of the 3D acceleration of each SCoM from MIMU data based on an inertial model;
- Expression and fusion of SCoM accelerations in a consistent common global frame *R_G_*;
- Estimation of the 3D BCoM acceleration and velocity from a weighted average of selected SCoM accelerations.

Since trunk, thighs, shanks and feet are the major contributors to 3D BCoM acceleration for people with transfemoral amputation [44], seven MIMUs were mounted on these segments and manually aligned with their respective longitudinal axes. Consistently with the cited reference, in the present work, the BCoM acceleration and velocity obtained from different combinations of sensors including from three to five of the abovementioned MIMUs were investigated with the proposed methodology (see Section 2.1.3). The next paragraphs describe the three steps of the framework in further detail, while its evaluation is detailed in Section 2.2.

#### 2.1.1. Computation of 3D SCoM Acceleration in the MIMU Local Frames

In a first step, 3D SCoM accelerations are computed from segment-mounted MIMUs in their respective MIMU local frame $R_{MIMU_{i}}$.

For each MIMU-bearing segment, the computation of SCoM acceleration ($a_{sCoM_{i}}$) from the acceleration measured at the origin of the MIMU ($a_{oIMU_{i}}$) is straightforward under the assumption that the SCoM and the MIMU are both rigidly attached to the same rigid body, and it only requires knowing the relative position between the SCoM and the MIMU origin (Equation (1)).

(1) $$a_{sCoM_{i}}=a_{oIMU_{i}}+\Omega_{IMU_{i}}\wedge \left(\Omega_{IMU_{i}}\wedge \;r_{IMU_{i}-sCoM_{i}}\right)+{\dot{\Omega }}_{IMU_{i}}\wedge r_{IMU_{i}-sCoM_{i}}\;\mathrm{in}\;R_{MIMU_{i}}$$

where $\Omega_{IMU_{i}}$ is the angular velocity measured by the MIMU, ${\dot{\Omega }}_{IMU_{i}}$ is the MIMU angular acceleration and $r_{IMU_{i}-sCoM_{i}}$ is the relative position between the MIMU origin and the SCoM expressed in $R_{MIMU_{i}}$.

However, it is not possible to directly obtain the position of a SCoM in its associated MIMU local frame. With the assumption that MIMUs are rigidly attached to their respective underlying segments, the relative position between each pair of SCoM and MIMU is constant in the MIMU local frame. Therefore, it is sufficient to determine this relative position at just one single instant in another reference frame, provided that the orientation of the MIMU local frame is known in this reference frame. Using an inertial model personalized to each participant with calibrated photographs, both the SCoM and the MIMU positions can be retrieved in a consistent reference frame corresponding to that of the photographs. The relative positions being expressed in the photograph frame, the orientation of each MIMU local frame in the photograph frame must be determined to express the relative position in the MIMU local frame and deduce the acceleration of each SCoM from Equation (1).

The present framework relies on a 15-segment subject-specific inertial model derived from Pillet and coworkers [46]. Similarly to [46], the photographs are calibrated using retro-reflective markers located on the ground and on the upper body. The positions of these markers are recorded with an OMCS while the photographs are being taken. For each MIMU, the position of its origin is then manually identified on the photographs, which allows computing its relative position with respect to the underlying SCoM ($r_{oIMU_{i}-sCoM_{i}}$) in the OMCS reference frame $R_{OMCS}$. Then, the transformation matrix $P_{OMCS-MIMU_{i}}$ from each MIMU local frame $R_{MIMU_{i}}$ to $R_{OMCS}$ must be known during the static acquisition. While the global frame $R_{GF_{i}}$ sensed by each MIMU has its vertical axis $\left(z_{GF_{i}}\right)$ coincident with that of the OMCS, each MIMU global frame and the OMCS frame may have a different heading due to perturbations of the magnetic field [29,30,47]. Consequently, the orientation output provided by each MIMU $P_{GF_{i}-MIMU_{i}}$ cannot be directly used to estimate the transformation matrix $P_{OMCS-MIMU_{i}}$ from $R_{MIMU_{i}}$ to $R_{OMCS}$ during the static phase. Instead, the framework relies on the knowledge of the manual alignment of MIMUs with the OMCS frame during the initial static posture. 

**Figure 1:**
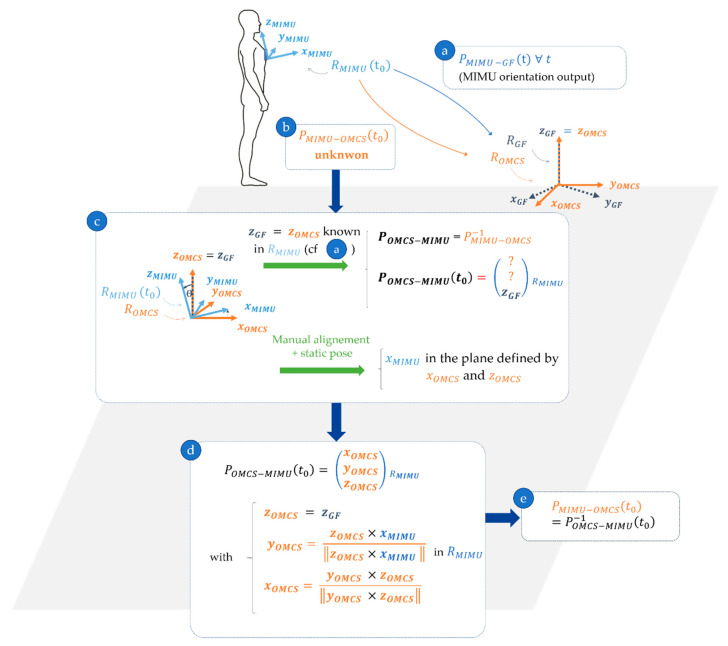
Computation of the orientation of the trunk MIMU local frame in the OMCS reference frame $P_{MIMU-OMCS}$ during the static posture (at $t=t_{0}$). To determine the orientation matrix, the axes of the OMCS reference frame must be determined in the MIMU local frame. $P_{MIMU-GF}$ is retrieved from the orientation output of the MIMU at $t=t_{0}$ (**a**). $P_{MIMU-OMCS}$ is unknown at $t=t_{0}$ (**b**) but it might be approximated using (**c**). Using the orientation output of the MIMU, the vertical direction $z_{GF}$ of the MIMU-sensed Earth-fixed frame is known in $R_{MIMU}$. Furthermore, since MIMUs’ attitude is not affected by magnetic perturbations, the vertical direction detection by MIMUs is robust and is consistent with that of the OMCS global frame $R_{OMCS}$. Therefore, $z_{GF}=z_{OMCS}$ in $R_{MIMU}$. The manual alignment of the MIMU on body segments and the static posture taken by the participant allows assuming that the x axis of the MIMU local frame $x_{MIMU}$ is in the plane defined by $x_{OMCS}$ and $y_{OMCS}$ axes. This in turn can be used to approximate $x_{OMCS}$ and $y_{OMCS}$ in $R_{MIMU}$ (**d**). Lastly, $P_{MIMU-OMCS}$ is obtained at $t=t_{0}$ as the inverse of $P_{OMCS-MIMU}$ (**e**).

The static posture in which the participant is being photographed has been defined such that he/she is standing facing the direction of progression. It is assumed that, in this position, each MIMU is aligned such that one of the axes of the MIMU local frame lies in the sagittal plane of the participant (which coincides with that of the OMCS frame—Equation (2)). Under this assumption and considering that (1) the vertical axis of the MIMU global frame coincides with that of the OMCS (Equation (3)) and (2) the orientation of the MIMU local frame in its global frame is known, it is then possible to express the orientation of the OMCS reference frame in the MIMU local frame $P_{OMCS-MIMU_{i}}$ during the static acquisition (Equations (4)–(7)). Figure 1 details the procedure for a MIMU positioned at the trunk level.

(2) $$x_{MIMU_{i}}\;\in \;\left\{x_{OMCS}\;;z_{OMCS}\right\}$$

(3) $$z_{OMCS}=z_{GF}\;\mathrm{known}\;\mathrm{in}\;R_{MIMU_{i}}$$

From Equations (2) and (3):

(4) $$x_{OMCS}\;\in \;{\left\{x_{MIMU_{i}}\;;z_{GF}\right\}}_{R_{MIMU_{i}}}$$

Then, $y_{OMCS}$ is orthogonal to the plane defined by ${\left\{x_{MIMU_{i}}\;;z_{GF}\right\}}_{R_{MIMU_{i}}}$ and

(5) $$y_{OMCS|R_{MIMU_{i}}}=\frac{z_{GF|R_{MIMU_{i}}}\times x_{MIMU_{i}|R_{MIMU_{i}}}{}_{\;}}{\left||z_{GF|R_{MIMU_{i}}}\times x_{MIMU_{i}|R_{MIMU_{i}}}|\right|}$$

(6) $$x_{OMCS|R_{MIMU_{i}}}=y_{OMCS|R_{MIMU_{i}}}\times z_{GF|R_{MIMU_{i}}}$$

(7) $$P_{OMCS-MIMU_{i}}={\left(\begin{array}{c} \begin{array}{c} x_{OMCS}\\ y_{OMCS} \end{array}\\ z_{OMCS} \end{array}\right)}_{R_{MIMU_{i}}}$$

The transformation matrix $P_{OMCS-MIMU_{i}}$ obtained during the static phase allows expressing the vector $r_{IMU_{i}-sCoM_{i}}$ obtained in the OMCS frame $R_{OMCS}$ in the MIMU local frame $R_{MIMU_{i}}$. It follows that SCoM accelerations can be computed in their respective sensor frame at all timestamps following Equation (1), where ${\dot{\Omega }}_{IMU_{i}}$ is obtained using a 5-point stencil differentiation of the angular velocity $\Omega_{IMU_{i}}$.

#### 2.1.2. Merging SCoM Accelerations in a Consistent Common Global Frame

**Figure 2:**
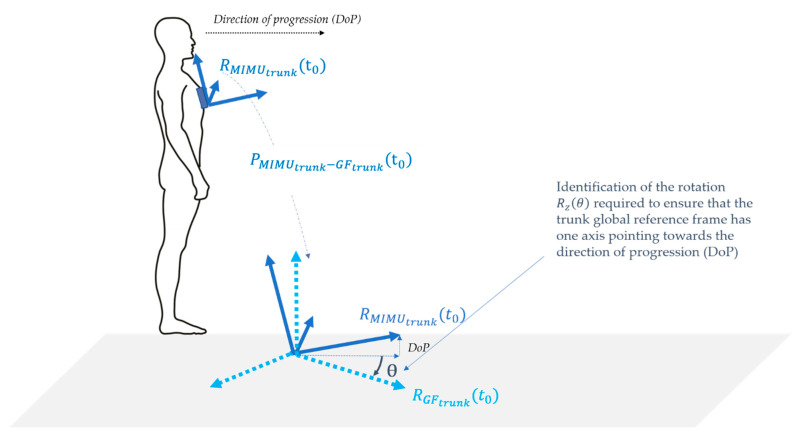
Rotation $R_{z}\left(\theta \right)$ of the trunk-MIMU-sensed Earth-fixed frame ($R_{GF_{trunk}})$ to align one of its axes with the direction of progression, using the orientation of the trunk MIMU local frame ($R_{MIMU_{trunk}}$).

Since MIMUs may sense inconsistent global frames ($R_{GF}$) [29], a consistent common global reference frame $R_{G}$ must be defined consistently for all MIMUs in order to merge the SCoM accelerations in a global reference frame and to compute the BCoM acceleration. In the present study, the reference frame sensed by the trunk MIMU $R_{GF_{trunk}}$, rotated so that one axis is coincident with the direction of progression, is chosen as the common global reference frame ($\;R_{G}=R_{z}\left(\theta \right)\times R_{GF_{trunk}}$, see Figure 2). This choice is supported by the lesser exposition of the trunk MIMU to magnetic perturbations compared to the MIMUs located on other segments, as the trunk lies farther from the ground [30] and is subject to low height variation while walking [48]. The direction of progression can be inferred from the orientation output of the trunk MIMU using the fact that one of its axes lies in the sagittal plane of the participant and is, therefore, oriented towards the direction of progression.

For each MIMU*_i_*, the constant transformation matrix $P_{G-GF_{i}}$ between the MIMU’s sensed global frame $R_{GF_{i}}\;$ and the common global reference frame $R_{G}=R_{z}\left(\theta \right)\times R_{GF_{trunk}}$ is obtained during the initial static posture at the beginning of each acquisition using the known orientation in $R_{OMCS}$ of both the trunk MIMU $\left(P_{OMCS-MIMU_{trunk}}\right)$ and MIMU*_i_* $\left(P_{OMCS-MIMU_{i}}\right)$ (Section 2.1.1), as well as their known orientation outputs ($P_{MIMU_{trunk}-GF_{trunk}},\;P_{MIMU_{i}-GF_{i}})$ (Equations (8)–(10)):

(8) $$P_{G-GF_{trunk}}=R_{z}\left(\theta \right)$$

(9) $$P_{G-GF_{i}}=P_{G-GF_{trunk}}\times P_{GF_{trunk}-GF_{i}\;}=R_{z}\left(\theta \right)\times P_{GF_{trunk}-GF_{i}\;}$$

(10) $$P_{G-GF_{i}}=R_{z}\left(\theta \right)\times P_{GF_{trunk}-MIMU_{trunk}}\left(t_{0}\right)\times P_{MIMU_{trunk}-OMCS\;}\left(t_{0}\right)\times P_{OMCS-MIMU_{i}}\left(t_{0}\right)\times P_{MIMU_{i}-GF_{i}}\left(t_{0}\right)$$

Using the constant transformation matrix $P_{G-GF_{i}}$ and the orientation output provided by each MIMU ($\;P_{MIMU_{i}-GF_{i}}=P_{GF_{i}-MIMU_{i}}^{-1}{}_{\;}$), the acceleration of each SCoM can be expressed in a consistent global reference frame at all timestamps:

(11) $$a_{sCoM_{i}}^{G}\left(t\right)=P_{G-GF_{i}}\times P_{GF_{i}-MIMU_{i}\;}\left(t\right)\times a_{sCoM_{i}}^{MIMU_{i}}\left(t\right)$$

#### 2.1.3. Estimating 3D BCoM Acceleration and Velocity 

##### Selected Sensor Networks

As mentioned above, based on the results of a previous work that analyzed the contributions of the body segments to the BCoM acceleration in ten people with transfemoral amputation [44], three sensor networks each composed of 3 to 5 segments were considered as good candidates for the estimation of BCoM acceleration and velocity (Table 1). BCoM acceleration and velocity obtained using a unique MIMU at the trunk level were also analyzed to verify the hypothesis that using multiple sensors instead of a single sensor would improve the accuracy of the estimates.

##### 3D BCoM Acceleration

For each of the selected sensor networks, SCoM accelerations of the included segments were expressed in $R_{G}$ and fused to compute 3D BCoM acceleration, with $m_{Seg_{i}}$ representing the mass of the $ith^{}$ segment derived from the personalized inertial model and N representing the number of MIMU-bearing segments included in the network (Equation (12)):

(12) $$a_{BCoM}=\overset{N}{\underset{i=1}{\sum }}\frac{m_{seg_{i}}}{\sum_{j=1}^{N}m_{seg_{j}}{}_{\;}}a_{SCoM_{i}}$$

##### 3D BCoM Velocity

The 3D BCoM velocity was computed stride per stride as the sum of the average walking speed and the cyclical component. Stride segmentation was performed at the prosthetic heel strike from shank MIMU readings [49,50]. Subsequently, the average component of 3D BCoM velocity (or “average walking speed”) was estimated as the ratio of the displacement of the prosthetic shank along the direction of progression within a stride to the stride duration, using the kinematic model specifically developed for people with lower-limb amputation by Durrafourg and coworkers [51]. The cyclical component of the 3D BCoM velocity was computed from direct numerical integration of 3D MIMU-based BCoM linear acceleration followed by high-pass filtering [52].

### 2.2. Evaluation of the Wearable Framework

#### 2.2.1. Experimental Protocol

**Figure 3:**
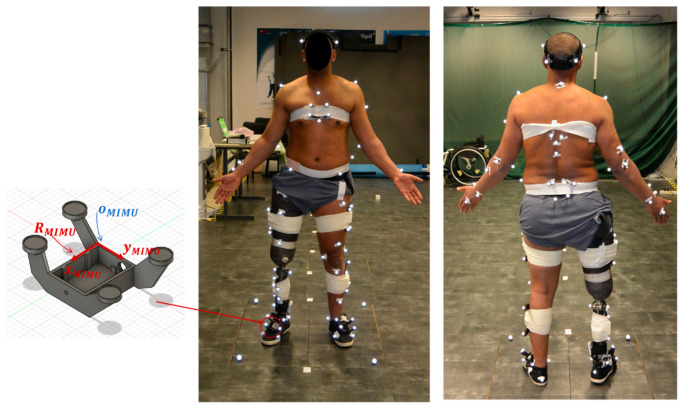
Full-body marker set and custom 3D-printed plastic MIMU housing.

A proof-of-concept validation was performed to evaluate the wearable-based framework. One male individual with transfemoral amputation (mass: 83 kg, stature: 1.69 m, age: 35 years old) gave his written informed consent to participate in the study, which was conducted according to the guidelines of the Declaration of Helsinki and approved by an independent Ethics Committee (Comité de Protection des Personnes, NX06036, approved on 1 March 2019). He was instrumented with a full-body marker set and seven MIMUs (Xsens Technologies B.V., Enschede, The Netherlands, 100 samples·s^−1^) on the feet, shanks, thighs and trunk, each mounted on a 3D-printed plastic support with housings for four reflective markers (Figure 3). An OMCS (Vicon, Oxford Metrics, UK, 200 samples·s^−1^) recorded the markers’ 3D position while four photographs (front, back, both sides) were taken. Then, starting from a static standing posture, the participant walked at self-selected speed along an 8 m pathway, with three force plates (AMTI, Advanced Mechanical Technology, Inc., Watertown, MA, USA, 1000 Hz) in the middle. Synchronization between instruments was achieved by an electronic trigger signal. Only trials with three successive foot contacts on the force plates (i.e., a complete stride), were considered for further analysis. 

#### 2.2.2. Data Processing

Data were filtered using a zero-phase fourth-order Butterworth filter. Cut-off frequencies were identified using a spectral analysis approach (5 Hz for marker and MIMU raw data, 10 Hz for force plates). Reference SCoM accelerations were obtained by double differentiation of OMCS-based SCoM positions. Each differentiation step was followed by a zero-phase low-pass Butterworth fourth-order filter with cut-off frequencies set to 8 Hz (velocity) and 10 Hz (acceleration). Reference 3D BCoM acceleration was computed from the force plates’ signal while reference 3D BCoM velocity was computed from the inertial model, to avoid error propagations due to ill-chosen integration constants when estimating the velocity from force platforms. 

For each sensor network configuration, reference and MIMU-based SCoM and BCoM accelerations/velocities were compared using Pearson’s correlation coefficient ρ, root mean square error (RMSE) and peak-to-peak normalized RMSE (NRMSE, as introduced in [53]) averaged over the trials. Errors in the estimation of BCoM velocity were also quantified in percentage of the average walking speed in the direction of progression (ARMSE). The average and standard deviation of the (normalized) RMSE respectively indicate the accuracy and precision of the methods.

## 3. Results

Seven trials, resulting in thirteen strides, were analyzed. Only the middle strides occurring entirely on the force plates were considered for the investigation of BCoM acceleration accuracy (i.e., seven strides), whereas the whole set of strides was analyzed for the SCoM acceleration and BCoM velocity.

### 3.1. SCoM and BCoM Acceleration

Results of the comparison between MIMU-derived and OMCS-based SCoM accelerations are provided in Table 2. Correlations between MIMU-based and reference SCoM acceleration were small at both feet and moderate at the sound shank in the mediolateral direction but were strong otherwise (ρ > 0.7).

**Figure 4:**
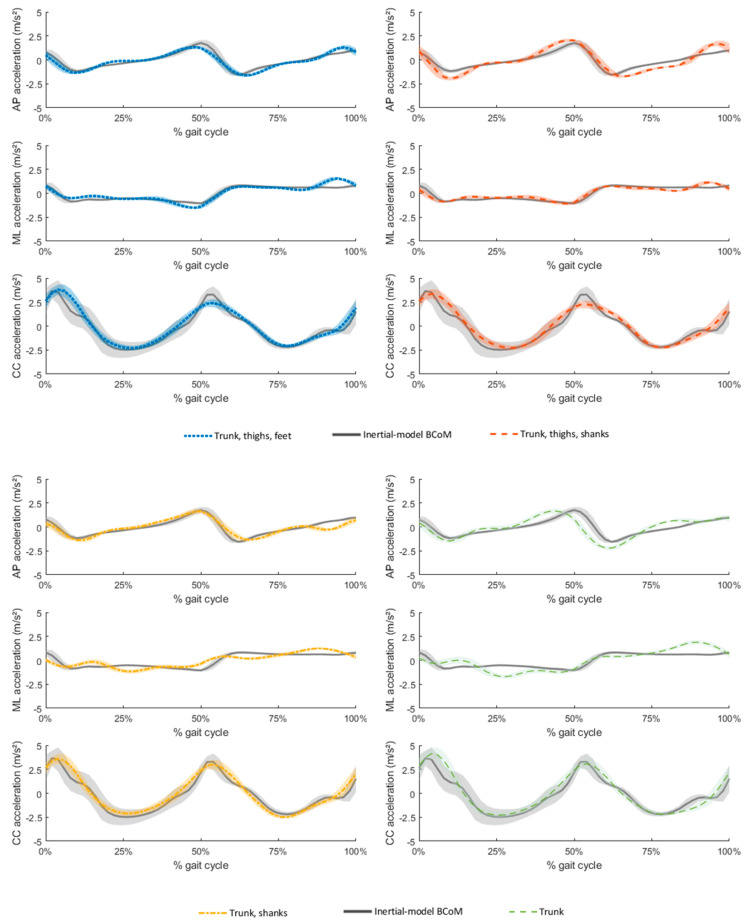
Acceleration of the body center of mass derived from force platform measures (gray straight line) and from the four different optimal sensor networks, consisting in the weighted sum of center of mass accelerations of the included segments (colored dashed and dotted lines), in the anteroposterior direction (AP), mediolateral direction (ML) and vertical direction (CC). Shaded regions represent the interval [mean − standard deviation; mean + standard deviation] for the estimates of the BCoM acceleration averaged over the 7 gait cycles of the participant.

Results of the comparison between MIMU-based and force-platform-based BCoM accelerations are provided in Table 3 and in Figure 4. Correlations between MIMU-based and reference BCoM acceleration were strong for all the tested sensor networks in all directions (ρ > 0.7). The added value of using multiple sensors instead of a single sensor at trunk level is demonstrated by the increased accuracy and the better fit of reference BCoM acceleration in the anteroposterior and mediolateral directions when using multiple-sensor networks (Table 3, Figure 4).

### 3.2. BCoM Velocity

**Figure 5:**
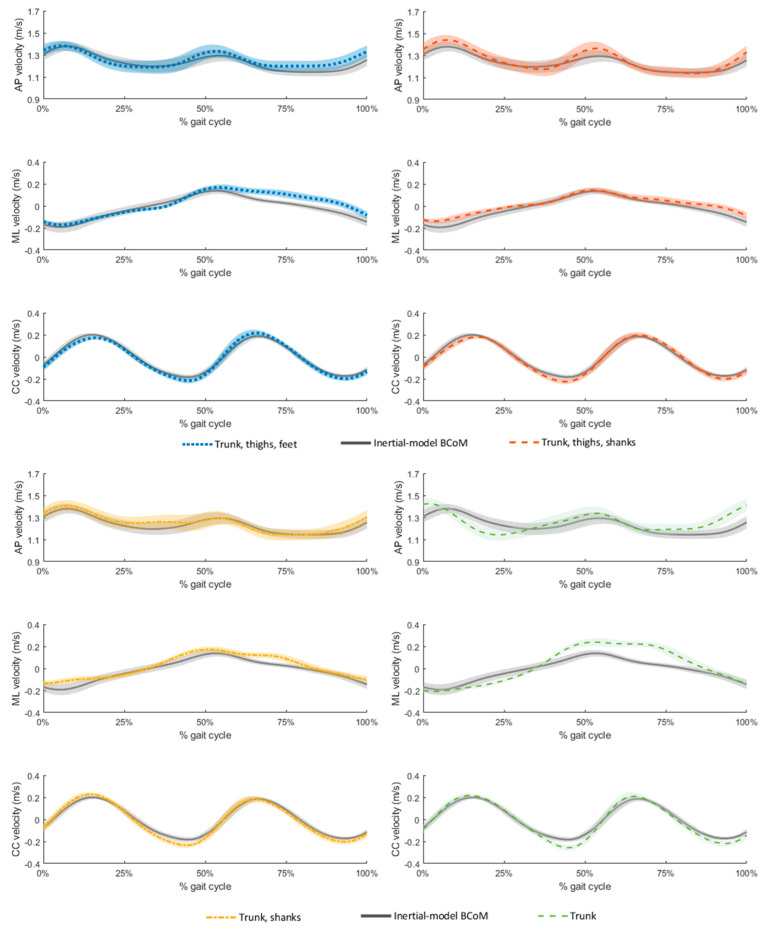
Body center of mass (BCoM) velocity as estimated by the selected sensor networks (upper-left corner (blue dotted lines): trunk, thighs, feet; upper-right corner (orange dashed lines): trunk, thighs, shanks; lower-left corner (yellow dashed lines): trunk and shanks; lower-right corner (green dashed lines): trunk) in comparison with the reference BCoM velocity obtained by optical motion capture (gray straight line). Shaded regions represent the interval [mean − standard deviation, mean + standard deviation] for each estimate of the BCoM velocity averaged over the thirteen prosthetic gait cycles of the participant in the anteroposterior (AP), mediolateral (ML) and vertical (CC) directions.

A comparison of the accuracy of sensor-network-based BCoM velocity to that of the reference inertial model is presented in Table 4. MIMU-based and reference BCoM velocity averaged over the thirteen prosthetic strides are displayed in Figure 5. Interestingly, the sensor networks that achieved the best estimation of BCoM velocity were different from those that achieved the best fit for BCoM acceleration. The five-MIMU sensor network including the shanks performed better than that including the feet in all directions, as displayed by the higher Pearson’s correlation coefficients and the lower RMSEs. BCoM velocity estimated with the trunk SCoM acceleration achieved a good fit of BCoM velocity with excellent correlations in the mediolateral and vertical direction (ρ ≥ 0.92), but only a moderate agreement in the anteroposterior direction (ρ = 0.57). Furthermore, high errors were recorded for this model in the anteroposterior and mediolateral directions (RMSE ≥ 0.08 m·s^−1^). 

## 4. Discussion

This study aimed at proposing and validating a framework for the estimation of both BCoM acceleration and velocity from an optimal network of MIMUs. Based on the results of an OMCS-based study performed on ten people with transfemoral amputation [44], several sensor networks were investigated, including from 3 to 5 MIMUs positioned on the trunk and on one or more pairs of the lower limb segments. The added value of using a multiple-sensor network instead of a single sensor at trunk level was also investigated by comparing the accuracy of the estimated quantities using the various sensor networks to that obtained with a single trunk-mounted MIMU. This pilot study demonstrated the feasibility of accurately estimating the 3D BCoM instantaneous walking velocity and acceleration for people with transfemoral amputation by using five MIMUs. The importance of this study resides in the lack of methods available to accurately estimate 3D BCoM kinematics from a limited number of sensors during gait, including for people with a lower-limb amputation. However, the fact that the framework was validated on one participant only should be kept in mind before generalization of the achieved results to the population of transfemoral amputees.

### 4.1. SCoM and BCoM Acceleration

In the developed framework, the BCoM acceleration is estimated through a weighted average of SCoM accelerations obtained from MIMUs. To the authors’ knowledge, this is the first study that reported accuracy results for the estimation of SCoM accelerations from MIMUs. 

Interestingly, when more than three sensors were used for estimating the BCoM acceleration, higher errors were recorded on average for the estimation of accelerations at the SCoMs than at the BCoM. Accelerations estimated at the shanks and feet had the highest errors and were poorly (sound limb) or moderately (prosthetic limb) correlated with the reference SCoM acceleration in the mediolateral direction. A possible reason for this discrepancy lies in the assumptions made regarding the alignment of the MIMU local frames with those of the global reference frame in static condition. Indeed, the participant was not specifically asked to stand with his feet parallel, which necessarily affected the hypothesis that one axis of the foot-mounted MIMU local frames lies in the sagittal plane. Natural outward alignment of the feet of 20° has been reported in the literature [54], which would have had an impact on the orientation of both the feet and the shanks. However, sensor networks that included feet and thigh segments were shown to be superior to their counterparts using shank-mounted MIMUs in terms of accuracy with the BCoM acceleration (Table 3).

In the present study, BCoM acceleration estimated using a single trunk-mounted sensor resulted in lower accuracy in the anteroposterior and mediolateral directions than that reported by Mohamed Refai and coworkers with a single MIMU at pelvis level in eight asymptomatic participants [35]. However, the presented framework achieved higher accuracy in the vertical direction and higher consistency with the reference acceleration pattern in the mediolateral and vertical directions, as demonstrated by higher correlation coefficients. When estimated using multiple sensors, MIMU-based BCoM acceleration results were in agreement with those reported in healthy subjects using OMCS-based accelerations [24]. Indeed, using three sensors (MIMUs mounted on the trunk and shanks), the method proposed in the present study achieved similar to improved accuracy (mean NRMSE) and better precision (standard deviation of the NRMSE) compared to the method proposed by Shahabpoor and coworkers using the acceleration of three different segments (trunk, pelvis and a thigh) in healthy participants (present study vs. healthy participants: 11.6 ± 2.1% vs. 16 ± 2.0% in the anteroposterior direction, 21.5 ± 2.7% vs. 18 ± 6.7% in the mediolateral direction and 7.7 ± 0.4% vs. 7 ± 1.7% in the vertical direction). Although walking variability for people with lower-limb amputation is higher with respect to typical gait [55], it may be speculated that the increased precision in the present study could be related to the fact that only intra-subject variability was considered since a single participant was tested. Conversely, six asymptomatic subjects ambulating at different walking speeds were recruited in [24]. It is worth noting that, in the former study, BCoM acceleration was estimated from a weighted average of SCoM accelerations derived from optical motion capture measurement. Therefore, decreased accuracy and precision are expected when transferring the methodology to MIMUs. The validity of the method presented in [24] when using wearable sensors was only investigated in the vertical direction, where a mean accuracy of 8.7% was achieved (1.7% decrease in accuracy). Therefore, the results achieved in the present study using a three-MIMU configuration can be considered very promising. 

Increasing the number of MIMUs allowed improving the accuracy of the estimated BCoM acceleration, particularly in the mediolateral direction (Table 3). Interestingly, the five-MIMU sensor network including sensors on the thighs and shanks resulted in an improved accuracy only in the mediolateral direction compared to the three-MIMU sensor network that did not include the thighs, while an increased accuracy in the anteroposterior direction was also observed when considering the five-MIMU network including the sensors on the thighs and feet. High consistency between reference and MIMU-based 3D BCoM acceleration patterns was observed with all the investigated sensor networks, with perceivable deviations in the mediolateral direction for the three-MIMU configuration (Table 3, Figure 4).

In light of these results, the three-segment sensor network including both shanks and the trunk appears to be optimal when the sagittal plane BCoM acceleration is targeted (anteroposterior and vertical directions). Differently, when the 3D BCoM acceleration must be estimated with high accuracy, a five-sensor model including the trunk, both thighs and either both feet or both shanks is to be preferred. 

### 4.2. BCoM Velocity

BCoM velocity was computed stride per stride using the sum of a cyclical component and an average component (average walking speed). The former was derived from the kinematic model developed in [51], which imposes the use of a MIMU mounted on the prosthetic shank, even when the sensor networks used for BCoM acceleration estimation did not include a sensor at the shank. In order to keep the number of sensors at a minimum, it is therefore preferred, with this integration method, to use sensor networks including the shank segments rather than the feet. On the other hand, the numerical integration of the foot acceleration between successive foot flat periods could be used to estimate the average walking speed [56]. However, reliable detection of foot flat events from inertial sensors may not be straightforward for people with lower-limb amputation.

BCoM velocity estimated from a single sensor at trunk level showed a slight phase anticipation in the anteroposterior direction (Figure 5) and lacked accuracy in the anteroposterior and mediolateral directions (average RMSE ≥ 0.08 m s^−1^) (Table 4). The use of multiple sensors arranged in networks allowed improving the estimated velocity by up to 13.2% in the anteroposterior direction. Interestingly, the three-MIMU sensor network including the trunk and shanks provided the most accurate estimate of BCoM velocity in the anteroposterior direction, with errors in the order of 3.0 ± 1.1% of the average walking speed (average RMSE = 0.04 ± 0.01 m s^−1^). Adding supplementary sensors at the thighs resulted in a better fit of the curve pattern in the anteroposterior and mediolateral directions (Figure 5), but it resulted in a slight decrease in accuracy in the anteroposterior direction (RMSE of 0.05 ± 0.02 m s^−1^, corresponding to 3.7 ± 1.0% of the average walking speed) due to the overestimation of BCoM velocity peaks in that direction (Figure 5). Therefore, although three MIMUs allowed estimating BCoM acceleration and velocity with a good accuracy index, using five MIMUs on the trunk, thighs and shanks should be preferred if a strong accuracy is required, especially in the mediolateral direction. The model including the foot sensors achieved lower accuracy in the anteroposterior and mediolateral directions than the models including the shanks. This might be a consequence of the assumption of parallel feet required for computing the relative orientation of the reference frames sensed by the feet MIMUs in the global reference frame (see Equation (2)).

As only a few studies in the literature have focused on the estimation of the instantaneous BCoM velocity, a direct comparison of our results with the existing literature is arduous. Furthermore, all former studies investigating the instantaneous BcoM velocity used the assumption that the BcoM was fixed in the pelvis anatomical frame. Sabatini and Mannini investigated a method for the estimation of the instantaneous velocity of a MIMU positioned at the pelvis compared to the velocity of an optical motion capture marker positioned on top of the MIMU [38]. Validation results are proposed separately for the cyclical component (limits of agreement (±1.96 standard deviation) of ±0.10 m s^−1^ in the anteroposterior and mediolateral direction and ±0.05 m s^−1^ in the vertical direction) and average component (RMSE = 0.07 m s^−1^ when ambulating overground). A smaller dataset was used in the present study, but higher accuracy was achieved for the cyclical component (±1.96 standard deviation of the RMSE: ±0.03 m s^−1^ in all directions, including when using a single sensor at the trunk level). 

Regarding the average walking speed, several authors have proposed algorithms for its estimation using inertial sensors [45]. Only two studies reported an estimate of the average walking speed within less than 3.7% of its nominal value. In [57], the average walking speed was estimated from double integration of the acceleration of a foot-mounted MIMU in 20 healthy participants (young and elderly) and achieved higher accuracy but lower precision (1.5 ± 5.8% of the actual walking velocity) than the proposed method. Using a shank-mounted MIMU and a kinematic model relying on stance knee flexion, which is absent for people with transfemoral amputation, Yang and coworkers estimated the average walking speed within 4.0% of its nominal value [58].

### 4.3. Limitations and Perspectives

The developed framework allowed the estimation of the instantaneous walking speed and acceleration of the body center of mass from five MIMUs positioned on the trunk and the thigh and shank segments of one person with transfemoral amputation. Results should be confirmed in a larger cohort prior to generalization. 

As a major requirement, the proposed wearable framework was designed to be as compatible as possible with clinical use. Currently, the framework requires the use of a camera and an optoelectronic system for the personalization of the geometric inertial model and the estimation of the relative position of each MIMU to the center of mass of the underlying segment in the intermediary photograph reference frame during a static acquisition. The use of these external devices, and especially of the optical motion capture system, compromises the direct transfer of the framework to the clinical field. The optical motion capture system was used for the calibration of photographs and for the construction of an initial geometric inertial model based on anatomical landmarks. Projections of the initial volumes on the frontal and sagittal photographs were manually reshaped so as to fit the participant’s body contours [46]. Therefore, using a different system for the calibration of photographs—or a method that does not require taking photographs at all—would facilitate the transfer of the framework to the clinical field. Regarding the first solution, the use of a device of known shapes and dimensions would allow calibrating photographs without the use of an OMCS. As for a possible alternative to taking photographs, body segmental inertial parameters and positions of anatomical landmarks and MIMUs could be retrieved from body meshes obtained with a 3D scanner. A semi-automatic method, requiring less than one minute of acquisition, has been proposed and validated in nine healthy participants [59]. Its validity for impaired people, in particular for people with a lower-limb prosthesis, remains to be verified. All in all, making the framework fully wearable does not appear to be a major issue, even if it would require some further development and validation. It should be noted that the method would still rely on an external portable device (camera/3D scanner) in order to retrieve the SCoM and MIMU positions in a consistent intermediary global frame (the scanner or camera frame). Therefore, the impact of errors in the estimations of the relative positions of MIMUs and SCoM on the output parameters (SCoM and BCoM acceleration, BCoM velocity) remains to be investigated. 

It should be noted that, in order to obtain the relative position of MIMUs and SCoM in the MIMU local frame, the framework uses a static calibration during which both the relative SCoM/MIMU positions and the orientations of MIMUs are estimated in an intermediary global frame. To do so, MIMUs were aligned such that one of the axes of their respective local frame lay in the sagittal plane of the motion. It should be stressed that this strong hypothesis was required only to derive the SCoM positions in their respective MIMU local frames and was not directly used for the definition of a consistent common reference frame or to derive segment orientation over time. Therefore, the impact of the misalignment of MIMUs on their respective underlying segment is believed to be minimal, which would not have been the case if the aim of this study was to derive joint angles [48,60]. Verification of this hypothesis should also be investigated in further studies.

The proposed framework could finally be enhanced in order to obtain complementary biomechanical parameters, such as individual limb ground reaction forces estimated from MIMUs [17,18]. For people with lower-limb amputation, in particular, receiving/giving feedback on the load distributed to each lower limb represents an interesting track for rehabilitation [61]. Several models proposing a smooth transition of the weight from one limb to another have been investigated in the literature [21,53], but their appropriateness for impaired gait remains to be assessed. Therefore, developing a method allowing the estimation of the ground reaction force under each foot from MIMU-based BCoM acceleration for people with transfemoral amputation represents a relevant track of research for future works. Furthermore, insight on mechanical energy exchanges can be inferred from the product of instantaneous BCoM velocity with the ground reaction force under each limb [8,62,63]. If data regarding the metabolic cost are available (possibly using regression equations from a body-worn accelerometer [64]), information about the actual patient’s locomotion efficiency can also be obtained. Such information about mechanical work, energy or efficiency can prove useful for the prescription of prosthetic components. An example in this field is provided by the work of Agrawal and colleagues [10], who proposed and validated an index based on the external work asymmetry between the sound and prosthetic limb to discriminate different prosthetic foot designs.

## 5. Conclusions

The results of the proposed framework are encouraging and suggest that MIMUs may represent a promising alternative to lab-based instruments when the 3D BCoM acceleration or velocity is targeted. Using a network of five MIMUs on the trunk, thighs and shanks indeed allowed the estimation of 3D BCOM acceleration and velocity in a person with transfemoral amputation with a strong agreement with reference data obtained from force platforms (acceleration: ρ ≥ 0.89) and an optical motion capture (velocity: ρ ≥ 0.94) with high accuracy (NRMSE ≤ 14% for the 3D acceleration and ≤ 15% for the 3D velocity). When only 2D BCoM kinematics are targeted, an inertial sensor network consisting of three MIMUs on the trunk and shanks was found to yield a similar level of accuracy and precision (ρ ≥ 0.89, NRMSE ≤ 13% for both 2D acceleration and 2D velocity). Results of this proof-of-concept study still need to be confirmed on a larger cohort to demonstrate the validity of MIMUs as an alternative motion capture system for 3D BCoM kinematics tracking. Furthermore, a test–retest design should be implemented to verify the inter-operator and intra-subject reproducibility of the method.

In the medium term, future studies will aim at assessing (1) the accuracy achieved when a fully wearable framework (that is, without an optical motion capture system) is implemented and (2) the impact of MIMU misplacement on the estimation of SCoM and BCoM kinematic parameters. 

## Figures and Tables

**Table 1:** List of the sensor networks investigated for the estimation of 3D BCoM acceleration and velocity using the wearable framework.

Number of Sensors	Instrumented Segments
5	Trunk, thighs, shanks
5	Trunk, thighs, feet
3	Trunk, shanks
1	Trunk

**Table 2:** Accuracy of segments’ center of mass accelerations estimated with MIMU compared to the optical motion capture reference in terms of root mean square error (RMSE), normalized RMSE and Pearson’s correlation coefficient (ρ). Means (standard deviations) over the considered stride cycles are reported.

Segment	RMSE (m·s^−^^2^)			NRMSE (%)			Pearson’s ρ		
	Anteroposterior	Mediolateral	Vertical	Anteroposterior	Mediolateral	Vertical	Anteroposterior	Mediolateral	Vertical
Prosthetic foot	2.94 (0.61)	2.74 (0.65)	2.00 (0.21)	5.2 (1.1)	26.1 (4.0)	6.6 (0.7)	0.97 (0.01)	0.27 (0.14)	0.96 (0.01)
Sound foot	3.64 (1.10)	3.99 (0.70)	3.31 (1.05)	6.3 (1.9)	22.1 (5.4)	8.4 (1.4)	0.96 (0.03)	0.19 (0.18)	0.90 (0.06)
Prosthetic shank	1.58 (0.33)	1.21 (0.39)	1.38 (0.08)	5.0 (1.0)	16.7 (5.3)	12.4 (0.8)	0.97 (0.01)	0.71 (0.16)	0.88 (0.02)
Sound shank	2.08 (0.43)	1.49 (0.43)	1.56 (0.19)	8.9 (1.6)	18.9 (4.1)	12.4 (1.9)	0.93 (0.03)	0.42 (0.20)	0.83 (0.05)
Prosthetic thigh	1.94 (0.07)	0.50 (0.11)	0.79 (0.02)	18.5 (0.6)	7.6 (1.7)	7.5 (0.4)	0.83 (0.03)	0.94 (0.04)	0.96 (0.00)
Sound thigh	2.10 (0.66)	0.72 (0.12)	0.94 (0.33)	10.5 (1.5)	14.6 (1.8)	9.5 (1.7)	0.85 (0.10)	0.74 (0.08)	0.90 (0.07)
Trunk	0.95 (0.05)	0.48 (0.04)	0.43 (0.22)	12.8 (1.1)	12.9 (1.1)	5.7 (2.4)	0.73 (0.04)	0.89 (0.02)	0.97 (0.03)
Average (all segments)	2.04 (0.99)	1.47 (1.25)	1.39 (0.95)	10.0 (4.6)	16.6 (6.3)	9.1 (2.8)	0.87 (0.10)	0.62 (0.30)	0.92 (0.06)

**Table 3:** Accuracy of sensor-network-based MIMU-derived BCoM acceleration as compared with force-platform-based acceleration in terms of root mean square error (RMSE), normalized RMSE and Pearson’s correlation coefficient (ρ). Means (standard deviations) over the considered stride cycles are reported.

Sensor Network	RMSE (m·s^−^^2^)			NRMSE (%)			Pearson’s ρ		
	Anteroposterior	Mediolateral	Vertical	Anteroposterior	Mediolateral	Vertical	Anteroposterior	Mediolateral	Vertical
Trunk, thighs, shanks	0.54 (0.02)	0.32 (0.03)	0.57 (0.06)	13.7 (0.9)	14.0 (2.1)	8.5 (0.5)	0.93 (0.01)	0. 89 (0.04)	0.95 (0.01)
Trunk, thighs, feet	0.33 (0.02)	0.37 (0.03)	0.51 (0.05)	9.7 (0.7)	13.7 (0.7)	7.4 (0.4)	0.93 (0.01)	0.88 (0.02)	0.96 (0.01)
Trunk, shanks	0.40 (0.06)	0.50 (0.05)	0.54 (0.04)	11.6 (2.1)	21.5 (2.7)	7.7 (0.4)	0.89 (0.03)	0.74 (0.08)	0.96 (0.00)
Trunk	0.66 (0.05)	0.70 (0.05)	0.63 (0.06)	17.0 (1.2)	23.5 (2.0)	8.8 (0.6)	0.78 (0.02)	0.76 (0.05)	0.95 (0.00)

**Table 4:** Accuracy of body center of mass (BCoM) velocity derived from the sensor-network-based BCoM acceleration compared to the reference velocity computed from optical motion capture in terms of root mean square error (RMSE), RMSE normalized to average walking speed (ARMSE) and peak-to-peak normalized RMSE (NRMSE).

Sensor Network	RMSE (m s^−1^)			ARMSE (%)	NRMSE (%)			Pearson’s ρ		
	Anteroposterior	Mediolateral	Vertical	Anteroposterior	Anteroposterior	Mediolateral	Vertical	Anteroposterior	Mediolateral	Vertical
Trunk, thighs, shanks	0.05 (0.02)	0.05 (0.01)	0.03 (0.02)	3.7 (1.0)	14.9 (4.2)	13.2 (3.0)	6.0 (0.8)	0.94 (0.04)	0.96 (0.03)	0.99 (0.00)
Trunk, thighs, feet	0.05 (0.01)	0.06 (0.02)	0.03 (0.01)	3.8 (0.8)	18.6 (5.3)	15.6 (3.9)	6.0 (0.6)	0.84 (0.05)	0.90 (0.04)	0.99 (0.01)
Trunk, shanks	0.04 (0.01)	0.05 (0.01)	0.03 (0.01)	3.0 (1.1)	13.2 (5.0)	13.7 (2.4)	6.7 (1.0)	0.92 (0.03)	0.94 (0.01)	0.99 (0.00)
Trunk	0.08 (0.01)	0.09 (0.01)	0.04 (0.01)	6.4 (0.6)	26.4 (2.8)	20.8 (1.7)	7.6 (0.8)	0.57 (0.06)	0.92 (0.02)	0.99 (0.00)
